# The ratio of T790M to EGFR-activating mutation predicts response of osimertinib in 1st or 2nd generation EGFR-TKI-refractory NSCLC

**DOI:** 10.1038/s41598-021-89006-9

**Published:** 2021-05-05

**Authors:** Motohiro Tamiya, Akihiro Tamiya, Norio Okamoto, Yoshihiko Taniguchi, Kazumi Nishino, Shinji Atagi, Tomonori Hirashima, Fumio Imamura, Toru Kumagai, Hidekazu Suzuki

**Affiliations:** 1 Department of Thoracic Oncology, Osaka International Cancer Institute, Chuo-ku Otemae 3-1-69, Osaka City, Osaka 541-8567 Japan; 2 Department of Internal Medicine, Kinki-Chuo Chest Medical Center, Kitaku Nagasone-cho 1180, Sakai City, Osaka 591-8555 Japan; 3 Department of Thoracic Malignancy, Osaka Habikino Medical Center, Habikino 3-7-1, Habikino City, Osaka 583-8588 Japan; 4 Department of Clinical Research Center, Kinki-Chuo Chest Medical Center, Kitaku Nagasone-cho 1180, Sakai City, Osaka 591-8555 Japan

**Keywords:** Cancer, Oncology

## Abstract

The most frequent mechanism of resistance after 1st/2nd-generation (G) epidermal growth factor receptor (EGFR)-tyrosine kinase inhibitors (TKIs) is secondary point mutation Thr790Met (T790M) in EGFR. Afatinib followed by osimertinib (Afa group) may provide better outcomes for T790M-positive non-small cell lung cancer (NSCLC) than 1st-G EGFR-TKI followed by osimertinib (1st-G group). We studied 111 consecutive NSCLC patients with T790M mutation treated with osimertinib after progression following 1st/2nd-G EGFR-TKI between March 28, 2016 and March 31, 2018. We analyzed the ratio of T790M to EGFR-activating mutation (T790M ratio) in post EGFR-TKI resistance re-biopsy tissue using droplet digital polymerase chain reaction. And investigated whether afatinib purified the T790M mutation more than 1st-G EGFR-TKI. Among 60 patients with preserved re-biopsy tissue, we analyzed 38 having adequate DNA content. The response rate in Afa group was 81.8% (n = 11) and 1st-G group was 85.2% (n = 27). The mean T790M ratio in total population was 0.3643. The ratio in those with response to osimertinib was significantly higher than in the non-responders (0.395, 0.202; *p* = 0.0231) and was similar in Afa and 1st-G group (0.371, 0.362; *p* = 0.9693). T790M ratio significantly correlated with osimertinib response and was similar between the 1st/2nd-G EGFR-TKIs in 1st/2nd-G EGFR-TKI-refractory tumors.

## Introduction

Based on randomized trials showing superior progression-free survival (PFS), response rate (RR), and more favorable safety profiles when compared with standard first-line platinum-based doublet chemotherapy in non-small cell lung cancer (NSCLC) patients with activating epidermal growth factor receptor (EGFR) mutation^1–6^, EGFR-tyrosine kinase inhibitors (TKIs), including gefitinib, erlotinib, and afatinib, have been established as the standard first-line treatment. However, cancer cells inevitably develop acquired resistance (AR) to EGFR-TKIs. Although EGFR-TKI treatment shows a durable response against NSCLC harboring *EGFR* mutations, most patients experience cancer relapse within 1–1.5 years following treatment with first-line 1st- and 2nd-generation (G) EGFR-TKIs. The most common resistance mechanism involves a secondary point mutation Thr790Met (T790M) in EGFR. It impairs the binding of TKIs to EGFR and is detected in approximately 50–60% of patients with 1st and 2nd-G EGFR-TKI-refractory tumors^7–9^. Osimertinib was developed for its activity against T790M by covalently binding to T790M-muted EGFR^10^, and it has been shown to be effective against AR of T790M-positive NSCLC after 1st- or 2nd-G EGFR-TKI treatment^11^. The RR in the trial was about 70% and use of osimertinib in 1st- and 2nd-G EGFR-TKI-refractory tumors was established as a standard treatment worldwide.

Moreover, osimertinib is associated with a longer PFS and overall survival (OS) than 1st-G EGFR-TKIs against advanced NSCLC harboring EGFR mutation (exon-19 deletion and L858R) as a first-line treatment^12^. However, in the Asian subset (especially in the Japanese subset) analysis of OS in the FLAURA study, osimertinib was not superior to 1st-G EGFR-TKIs^13^. There may be no molecular targets for therapy due to the heterogeneity of resistance mechanisms, which are not well understood^14,15^. As a result, during clinical care for most patients following cancer progression after osimertinib treatment, chemotherapy is the only remaining option for second-line treatment.

In contrast, afatinib demonstrated superior RR, PFS, and a trend of longer OS compared to gefitinib in the LUX-Lung 7^16^. A study showed that afatinib treatment followed by osimertinib indicated an extremely long treatment time to failure (TTF) and OS, even after removal of selection bias^17^. Furthermore, we reported that treatment with afatinib followed by osimertinib (Afa group) may provide better outcomes for T790M-positive NSCLC than that with 1st-G EGFR-TKIs (1st-G group)^18^. These results may be explained by the possibility that afatinib exhibits increased efficacy by targeting co-occurring EGFR mutations that enrich T790M cells.

Therefore, to investigate whether afatinib purifies T790M mutation more effectively than the 1st-G EGFR-TKIs, we analyzed the difference in the ratio of T790M mutation to EGFR-activating mutation ratio (T790M ratio) between the Afa and 1st-G group by using droplet digital polymerase chain reaction (ddPCR).

## Materials and methods

We conducted a multicenter-retrospective study across three medical centers (Osaka International Cancer Institute, Osaka Habikino Medical Center, and National Hospital Organization Kinki-Chuo Chest Medical Center) in Japan. The study design and methodology were approved by the Institutional Review Board of each participating institution (Institutional Review Board of Osaka International Cancer Institute, Institutional Review Board of Osaka Habikino Medical Center, and Institutional Review Board of National Hospital Organization Kinki-Chuo Chest Medical Center), and it was conducted in accordance with the Declaration of Helsinki and the World Health Organization’s Guidelines for Good Clinical Practice. We obtained the informed consent from participants and used an opt-out method so that patients and their families could refuse to participate in the study.

### Patient selection and ddPCR measurement

Between March 28, 2016 (the date osimertinib was approved in Japan) and March 31, 2018, study participants were consecutively enrolled according to the following criteria: patients with T790M mutation who were treated with osimertinib after AR to EGFR-TKIs at any time for advanced NSCLC, had good Eastern Cooperative Oncology Group (ECOG) performance status (PS): 0–2, and had major *EGFR* mutation (Exon-19: deletion19 or Exon-21: L858R) before initial EGFR-TKI treatment to reduce bias towards patient conditions. Furthermore, we excluded cases where we detected T790M mutation in the pre-treatment tissue by EGFR test in general practice.

We used RIKEN GENESIS CO., LTD. (Tokyo, Japan) to measure exon-19 deletion, L858R, and T790M by droplet digital polymerase chain reaction (ddPCR). The ddPCR analysis was performed using QX200 AutoDG Droplet Digital PCR System (BioRad). In this study, we excluded samples that had less than 1000 total copies/20 uL well or less than 10 copies with EGFR-activating mutation/20 uL well. T790M ratio was calculated by the following method. First, the fractional abundance (mutant allele/mutant allele + wild type allele) of activating mutation and T790M were measured with droplet count by ddPCR. Next, T790M ratio defined the ratio of T790M to activating mutation.

### Statistical analysis

We evaluated the systemic response to osimeritinib using the Response Evaluation Criteria in Solid Tumors (RECIST) ver1.1^19^. We used Fisher’s exact tests for categorical comparisons of data and compared differences in continuous data using the Wilcoxon test. Kaplan–Meier curves were used to evaluate PFS, which were compared using the log-rank test. Median values and 95.0% confidence intervals (CIs) were also reported. All statistical analyses were conducted using R software, version 2.8.1 (http://R-project.org) (The R Foundation for Statistical Computing, Vienna, Austria). *p* < 0.05 was considered a statistically significant difference.

## Results

**Figure 1:**
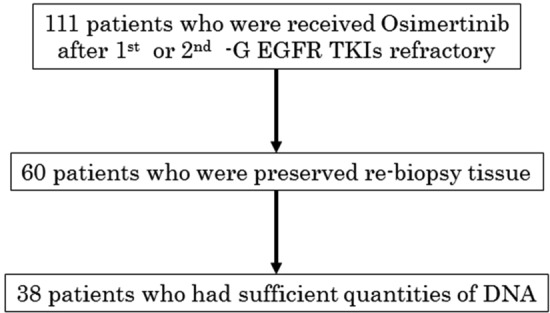
CONSORT diagram. 1st-and 2nd-G EGFR-TKIs: first and second generation epidermal growth factor receptor-tyrosine kinase inhibitors, *DNA* deoxyribonucleic acid.

Of the 111 enrolled patients, the re-biopsy tissue obtained from each patient before osimertinib treatment was preserved for 60 patients. Among them, 38 samples yielded enough DNA for ddPCR analysis, and the T790M ratio was assessed (Fig. 1). The patient characteristics are shown in Table 1. The median age was 68 years. Of the 38 patients, 21.1% were men, 23.7% had a history of smoking, 44.7% had L858R mutation, and 55.3% had exon 19 deletion. Eleven (28.9%) were in the Afa group and 27 (71.1%) were in the 1st-G group. The RR in all patients was 84.2%, 81.8% in the Afa group, and 85.2% in the 1st-G group (Table 2).

**Table 1:** Patient characteristics.

Characteristics	All patients n = 38
**Age**	
Median (range)	68 (41—86)
**Sex**	
Male/female	9/29
**Smoking history**	
Never/ever	17/21
**ECOG performance status**	
0/1/2	10/24/4
**Histology**	
Adenocarcinoma/others	38/0
**Sensitive EGFR mutation**	
19 deletion/L858R	20/18
**The last EGFR-TKI**	
1st-G EGFR-TKIs/Afatinib	27/11

1st-G EGFR-TKIs: 1st-generation EGFR-TKIs; *ECOG* Eastern Cooperative Oncology Group.

**Table 2:** The efficacy of osimertinib following between afatinib and 1st-G group.

	N	RR (%)	DCR (%)	CR (%)	PR (%)	SD (%)	PD (%)
All patients	38	84.2%	78.4%	0 0%	32 84.2%	3 7.9%	3 7.9%
Afatinib group	11	81.8%	100%	0 0%	9 81.8%	2 19.2%	0 0%
1st-G group	27	85.2%	88.9%	0 0%	23 85.2%	1 3.7%	3 11.1%

1st-G group: patients treated with 1st-generation EGFR-TKIs followed by osimertinib; afatinib group: the patients treated with afatinib followed by osimertinib.

*RR* response rate, *DCR* disease control rate, *CR* complete response, *PR* partial response, *SD* stable diseases, *PD* progression diseases.

**Figure 2:**
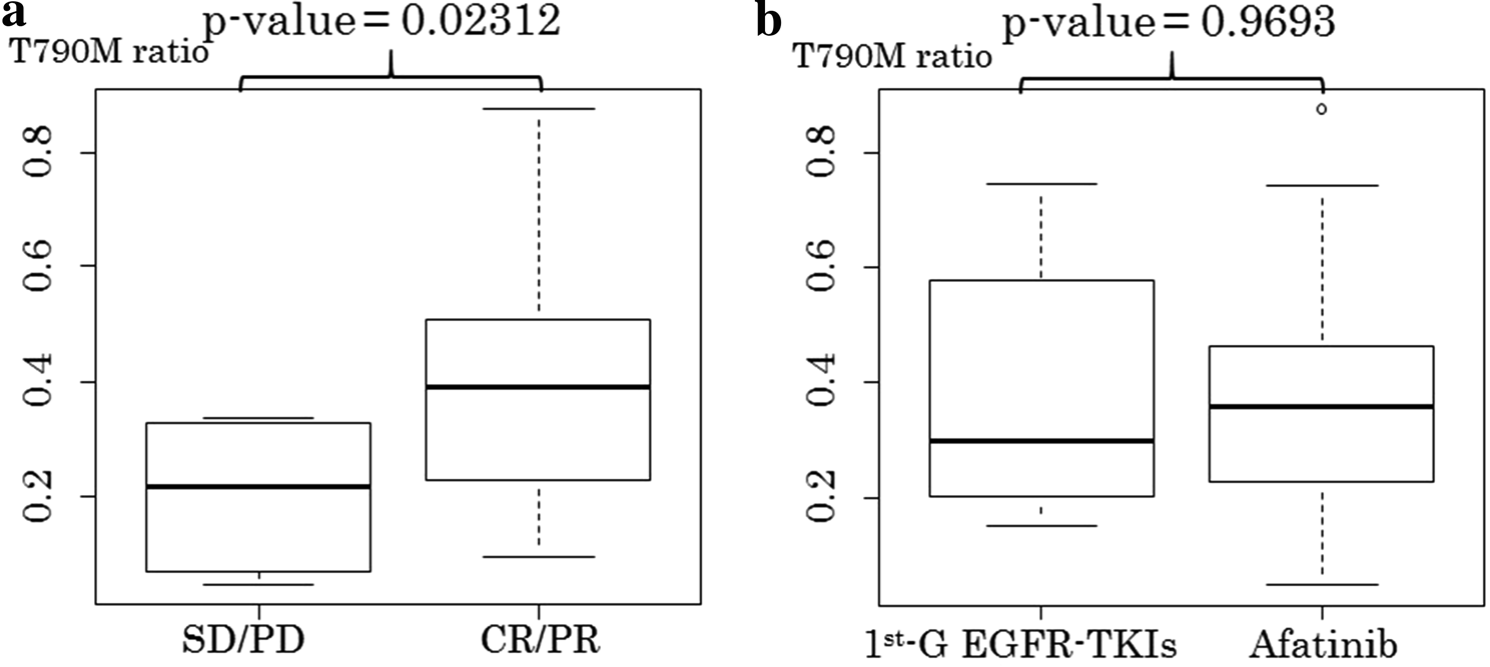
Box plot of the comparison with the T790M ratio. (**a**) SD/PD versus CR/PR, (**b**) 1st-G EGFR-TKIs versus Afatinib. *T790M ratio* T790M mutation to EGFR-activating mutation ratio, *CR* complete response, *PR* partial response, *SD* stable diseases, *PD* progression diseases, *1st-G EGFR-TKIs* first generation epidermal growth factor receptor-tyrosine kinase inhibitors.

The mean T790M ratio in the total population was 0.3643 (range: 0.0457–0.8774). The T790M ratio in patients who obtained complete response (CR) or partial response (PR) to osimertinib in the osimertininb group (mean: 0.395) was significantly higher than in patients with stable disease (SD) or progressive disease (PD) (mean: 0.202); *p* = 0.0231 (Fig. 2a). The T790M ratio in the Afa group (mean: 0.3711, range: 0.1497–0.7465) was the same with the 1st-G group (mean: 03616, range: 0.0457–0.8774); *p* = 0.9693 (Fig. 2b).

**Figure 3:**
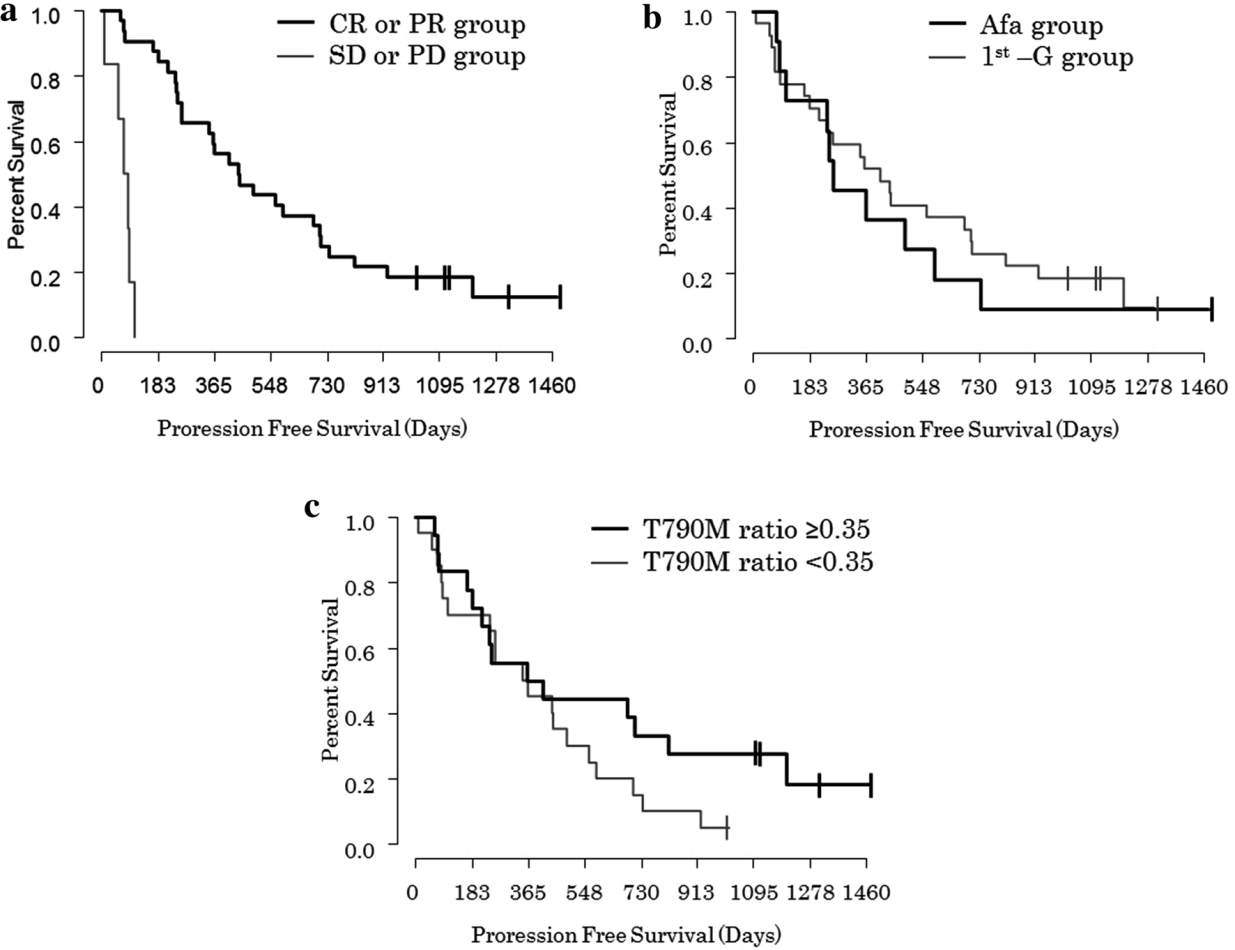
Kaplan-Meyer curves for progression free survival of osimertinib by (**a**) SD/PD versus CR/PR, (**b**) 1st-G EGFR-TKIs versus Afatinib, (**c**) T790M ratio ≥ 0.35 versus < 0.35. *CR* complete response, *PR* partial response, *SD* stable diseases, *PD* progression diseases, *1st-G EGFR-TKIs* first generation epidermal growth factor receptor-tyrosine kinase inhibitors, *T790M ratio* T790M mutation to EGFR-activating mutation ratio.

The median PFS in the CR or PR subgroup of the Osimertinib group (446 days [95% CIs 347–710]) was significantly longer than that in the SD or PD subgroup (77 days [95%CIs 52-not available (NA)]); *p* < 0.0001 (Fig. 3a). The median PFS in the Afa group (258 [95%CIs 239-NA] days) was also similar to the 1st-G group (414 days [95%CIs 241–710]); *p* = 0.6 (Fig. 3b).

**Figure 4:**
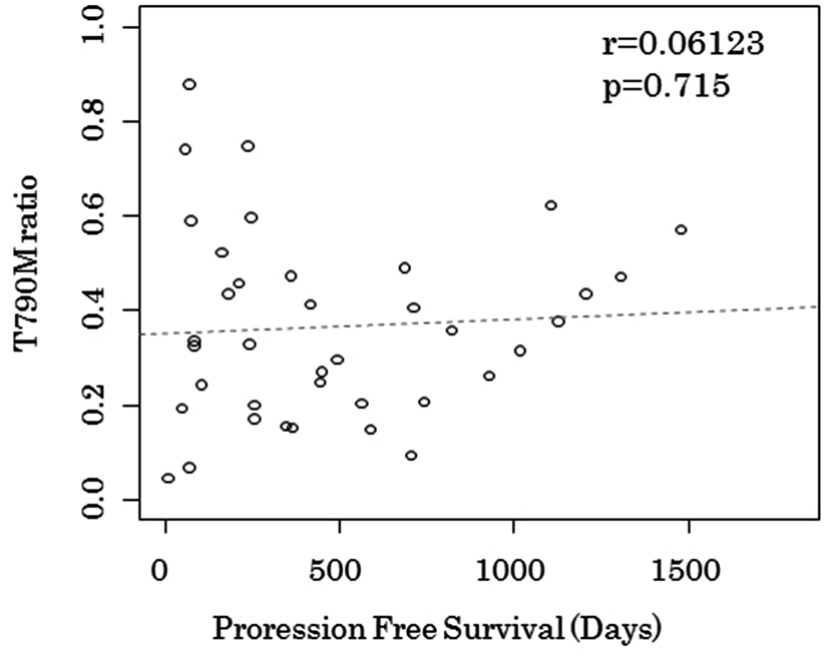
Scatter plot of between T790 ratio and progression free survival of osimertinib. T790M ratio, T790M mutation to EGFR-activating mutation ratio.

Furthermore, we divided two groups based on a threshold T790M ratio of 0.35 from the mean T790M ratio in total population. The response rate in patients with T790M ratio ≥ 0.35 (100% [18/18]) was higher than that in patients with T790M ratio < 0.35 (70% [14/20]); *p* = 0.03691. The median PFS in patients with T790M ratio ≥ 0.35 was longer when compared with patients with T790M ratio < 0.35 (387 days [95%CIs 214-NA] versus 356 days [95%CIs 241-NA]; *p* = 0.2), however this was not statistically significant (Fig. 3c). Pearson correlation analysis was performed to examine the relationship between T790M ratio and PFS. The analysis showed a correlation coefficient of r = 0.06123 (*p* = 0.715) (Fig. 4).

## Discussion

This is the first analysis to reveal a potential implication that afatinib purifies the T790M mutation more than the 1st-G EGFR-TKI. This study suggested that the T790M ratio in 1st/2nd-G EGFR-TKI-refractory tumors significantly correlated with response to osimertinib. The T790M ratio in CR or PR subgroup of the osimertinib group (mean: 0.395) was significantly higher than in the SD or PD subgroup (mean: 0.202); *p* = 0.0231. However, we did not observe a difference in the T790M ratio between the 1st- and 2nd-G EGFR-TKIs. The T790M ratio in the Afa group (mean: 0.3711) was similar to the 1st-G group (mean: 03616); *p* = 0.9693.

Osimertinib was developed as a drug against T790M that works by covalently binding to T790M-muted EGFR^10^. Therefore, although osimertinib is also effective against sensitive EGFR mutations, the most effective targets are T790M-positive EGFR mutations. In contrast, afatinib irreversibly binds to EGFR, ERBB2, and ERBB4 and blocks transphosphorylation of ERBB3 to inhibit all ERBB family signaling^20^. Two hypotheses were proposed: the first is that the broader inhibition of the EGFR family may improve the efficacy of EGFR-TKIs for NSCLC with EGFR mutation, and the second is that the broader inhibition of the EGFR family may attenuate T790M in cancer cells during relapse after EGFR-TKI treatment. Osimertinib could also be more effective against the AR of T790M after EGFR-TKI. The results of LUX-Lung7 and ARCHER1050 supported this theory in the first-line treatment for NSCLC with EGFR mutation^16,21^. Furthermore, afatinib provided the efficacy for uncommon EGFR mutations in preclinical and clinical studies^22,23^. In the analysis of GIO-TAG trials, the duration of response to osimertinib in patients receiving afatinib and subsequent osimertinib was highly encouraging^17,24,25^.

In our study, the concentration of T790M correlated with the response to osimertinib, and our results suggested that the degree of attenuation of T790M is important in eliciting the effects of osimertinib. Some prior studies have also shown similar results: the ratio of T790M to EGFR-activating mutation in cell samples or plasma samples may predict the response to osimertinib or rociletinib^26–28^.

However, we did not observe differences in the clonality of T790M after AR between afatinib and 1st-G EGFR-TKI. One reason was that the response of osimertinib after AR was the same in Afa group and 1st-G group when we collected the available tissue, although our previous study revealed that the objective RR and disease control rate were significantly higher in the Afa group than in the 1st-G group^18^. We intended to collect all the samples in our previous study; however, this was not possible. Another reason is that later lines of therapy may have advanced clonal evolution and may not reflect the effect of osimertinib after 1st-G EGFR-TKI and afatinib treatment.

Next, Pearson correlation analysis showed no significant relationship between T790M ratio and PFS of osimertinib after AR of EGFR-TKIs (r = 0.06123, *p* = 0.715). We believed that the PFS of osimertinib after AR in NSCLC with T790M mutation was influenced by co-mutation.

Based on clinal evolution, which was reported for the first time as early as 1976^29^, lung adenocarcinoma seems to follow a multistep progression from atypical adenomatous hyperplasia to adenocarcinoma in situ, and finally invasive adenocarcinoma^30^. EGFR driver alterations are acquired in the early step of cancer progression and can be identified in most neoplastic cancer cells^31^. Furthermore, by layering exacerbations of disease after treatment, especially EGFR-TKI use, cell progression will show different genomic patterns of selection through different lines of therapy^32^. In this setting, EGFR-sensitive mutant tumor cells may coexist with sub-clonal tumor cells harboring other gene mutations, and co-mutations such as TP53 affect the efficacy of osimertinib^33,34^. Furthermore, histologic transformation and other off-target molecular alterations are frequent early emerging resistance mechanisms to osimertinib and are associated with poor clinical outcomes^35^.

We must consider some limitations of the present study. First, this was a retrospective study, and the participants were selected based on obtainment of adequate samples. Second, the line of treatment for EGFR-TKIs was not determined. Since the lines of EGFR-TKIs varied from case to case, the effectiveness of osimertinib may also vary accordingly. Although osimertinib response was not different between the two groups, we focused on the attenuation of tumor cells by EGFR-TKIs immediately before the administration of osimertinib to clarify the correlation between T790M ratio and osimertinib response. Third, in this study, we examined only exon-19 deletion, L858R and T790M by ddPCR and did not perform next generation sequencing. Therefore, we cannot know about the presence of other EGFR mutations, amplifications, alternative bypass pathways or co-mutations that may provide a mechanism of resistance to osimerutinib.

## Conclusion

In our analysis, the T790M ratio in 1st/2nd-G EGFR-TKI-refractory tumors significantly correlated with osimertinib response. Furthermore, the T790M ratio between the 1st- and 2nd-G EGFR-TKIs was almost similar in patients with EGFR-TKI-refractory NSCLC who showed response to osimertinib. Further analysis in a larger number of prospective studies is warranted to confirm our results.
